# Hyperplasia of Pericytes Is One of the Main Characteristics of Microvascular Architecture in Malignant Glioma

**DOI:** 10.1371/journal.pone.0114246

**Published:** 2014-12-05

**Authors:** Huiqin Sun, Deyu Guo, Yongping Su, Dongmei Yu, Qingliang Wang, Tao Wang, Qing Zhou, Xinze Ran, Zhongmin Zou

**Affiliations:** 1 Institute of Combined Injury, State Key Laboratory of Trauma, Burns and Combined Injury, College of Preventive Medicine, Third Military Medical University, Chongqing, China; 2 Institute of Pathology and Southwest Cancer Center, Southwest Hospital, Third Military Medical University, and Key Laboratory of Tumor Immunopathology, Ministry of Education of China, Chongqing, China; 3 Institute of Toxicology, College of Preventive Medicine, Third Military Medical University, Chongqing, China; University of Torino, Italy

## Abstract

**Objectives:**

To investigate the role of pericytes in constructing the malformed microvessels (MVs) and participating microvascular architecture heterogeneity of glioma.

**Methods:**

Forty human glioma tissue samples (WHO grade II-IV) were included in present study. Observation of blood vessel patterns, quantitative analysis of endothelial cells (ECs)- and pericyte-labeled MVs and comparison between malignant grades based on single- or double-immunohistochemical staining. The MV number density (MVND), microvascular pericyte number density (MPND), and microvascular pericyte area density (MPAD) were calculated. The expression of PDGFβ was also scored after immunostaining.

**Results:**

In grade II glioma, most of tumor MVs were the thin-wall CD34^+^ vessels with near normal morphology. In addition to thin-wall CD34^+^ MVs, more thick-wall MVs were found in grade III glioma, which often showed α-SMA positive. Most of MVs in grade IV glioma were in the form of plexus, curled cell cords and glomeruloid microvascular proliferation while the α-SMA^+^ cells were the main components. The MVs usually showed disordered arrangement, loose connection and active cell proliferation as shown by Ki67 and α-SMA coexpression. With the increase of glioma grades, the α-SMA^+^ MVND, CD34^+^ MVND and MPND were significantly augmented although the increase of CD34^+^ MVND but not MPAD was statistically insignificant between grade III and IV. It was interesting that some vessel-like structures only consist of α-SMA^+^ cells, assuming the guiding role of pericytes in angiogenesis. The expression level of PDGFβ was upregulated and directly correlated with the MPND in different glioma grades.

**Conclusion:**

Hyperplasia of pericytes was one of the significant characteristics of malignant glioma and locally proliferated pericytes were the main constituent of MVs in high grade glioma. The pathological characteristics of pericytes could be used as indexes of malignant grades of glioma.

## Introduction

Microvasculature, one of the important parts of tumor stroma, is formed by angiogenesis, which termed the sprouting or splitting of vascular cells from pre-existed vessels of surrounding tissues. Since Folkman's first suggestion that new blood vessel formation was necessary for tumor growth and metastasis, it has been accepted that angiogenesis plays a key role in tumor progression [1], [2]. The great progress that achieved in the mechanism studies of tumor angiogenesis has dramatically promoted the therapeutic application of anti-angiogenesis strategy in tumor treatment [2]–[5]. Inspired by its promising results in clinical and experimental studies, many efforts have been made to elucidate the mechanism of tumor angiogenic regulation and to test the efficacy of different anti-angiogenic drugs. Recent research results from our and other's studies [6]–[9] have suggested that the microvasculature of tumor may vary with tumor types, heterogeneity of tumor cells and tumor stages. This may present the multiplicity and variability of tumor microvasculature, which subsequently affect tumor growth, treatment response and prognosis [10]. Therefore, tumor microvascular heterogeneity not only adds a new concept to oncology but also highlights a new research field [10], [11]. Further study on the characteristics of tumor microvasculature heterogeneity and its relationship with tumor types will help us to understand the role of microvasculature in oncobiology.

Malignant glioma is known to be a highly lethal type of tumors with the most active angiogenesis ability and variform vascular morphology in solid tumors [12], and is one of the best models for studying angiogenesis and antiangiogenesis [13], [14]. Actually, there are two main types of cells that constitute the microvessels (MVs), the liner is a monolayer of endothelial cells (ECs) and around outsides the pericytes. Both of these two types of cells are involved in angiogenesis by differentiation, proliferation, migration and mutual interaction [15]. But, for quite a long time, researchers only focus on ECs and neglect the existence of pericytes. With the discovery and its application of pericyte markers, the role of pericytes in vasculogenesis, angiogenesis and vessel remodeling have been gradually unveiled, and more attentions have been paid to explore the important or even the leading role of pericytes in angiogenesis [15]–[19].

In previous study, we found that the expression of endothelial marker did not consistently upregulated with the increase of tumor grade in multiform hyperplastic vasculatures of glioma, especially in the forms of thick-wall vessels or glomeruloid microvascular proliferation. This data conveys a critical message that hyperplastic changes in glioma vasculature maybe induced by another component of MV wall, the pericytes, rather than endothelial cells [11], [20], [21]. Present research analyzed the constituent pericytes in vasculatures of glioma in different grades of glioma, and studied the relationship between pericytes and tumor microvascular architecture heterogeneity. The vascular architecture of human glioma tissue was investigated by immunostaining the markers of ECs and pericytes. The location, pattern and distribution of pericytes in human glioma and their relationship with microvascular architecture in different grade (WHO grade II, III and IV) of glioma was assessed. The CD34^+^ or α-SMA^+^ MV number density (MVND), microvascular pericyte number density (MPND), α-SMA^+^ microvascular pericyte area density (MPAD), MV proliferation status and the expression of PDGFβ/PDGFR-β were also analyzed. The results demonstrated the important role of pericytes in the microvascular architecture heterogeneity of glioma.

## Materials and Methods

### Cases and samples

All the study protocols were reviewed and approved by the Ethics Committee of the Southwest Hospital, Third Military Medical University (TMMU), Chongqing, China. All patients were notified and their written informed consents were obtained. Formalin-fixed paraffin-embedded tissue samples from 40 patients with different grade glioma (astrocytoma) were collected from Institute of Pathology, Southwest Hospital in the period of 2006-2009. The written reports of all patients with glioma were reviewed and all cases in the classification of WHO grade II, III, and IV were collected according to the 2007 WHO classification of central nervous system tumors [22]. Pathological sections were reviewed by at least two experienced pathologists (H.S, D.Y and D.G), and the pathological diagnosis were reassessed based on the current World Health Organization Classification of Tumors as WHO grade II (n = 12), III (n = 12) and IV (Glioblastoma multiforme, GBM, n = 16).

### Tumor histology and microvascular morphology observation with H&E staining

Formalin-fixed, paraffin-embedded samples were cut into 4 µm-thickness sections and subjected to routine H&E staining. The sections were read by at least two pathologists with expertise, and the histopathological features and microvascular architectures of glioma were recorded and analyzed.

### Immunohistochemical staining

Following the instruction of the SP system kit (Zhongshan Golden Bridge Biotechnology Co., Ltd, Beijing, China), the 4 µm-thickness serial sections were mounted on aminoacetylsilane-coated slides (Zhongshan Golden Bridge Biotechnology Co., Ltd, Beijing, China) and subjected to standard immunohistochemical staining. The primary antibodies were incubated in a moist chamber overnight at 4°C to detect CD34 (mouse anti-human monoclonal antibody, clone QBEnd10, 1∶50 dilution, Dako, Copenhagen, Denmark), α-SMA (mouse anti-human monoclonal antibody, clone 1A4, 1∶100 dilution, Dako, Copenhagen, Denmark), PDGFβ (rabbit anti-human polyclonal antibody, 1∶100 dilution, Santa Cruz Biotechnology Inc., Santa Cruz, CA, USA), PDGFR-β (rabbit anti-human polyclonal antibody, 1∶100 dilution, Santa Cruz Biotechnology, Santa Cruz, CA, USA), Ki67 (mouse anti-human monoclonal antibody, clone SP6, 1∶100 dilution, Thermo Fisher Scientific Inc. IL, USA). After washing with PBS, the corresponding secondary antibody reaction was conducted. Diaminobenzidine tetrachloride from the SP kit was used as the chromogen for visualization. Slides were counterstained with hematoxylin, dehydrated, clarified and finally coverslipped. Negative controls consisted of incubating normal mouse/rabbit IgG replacement of the primary antibody.

For the double immunohistochemical staining, the DouSP Kit (Maixin Biotechnology Co., Ltd, Fuzhou, China) was used. The immunohistochemical double staining against CD34 and α-SMA, and α-SMA and Ki67 were performed according the user's manuals. The primary antibodies for CD34 (clone QBEnd10), α-SMA (clone 1A4) and Ki67 (clone SP6) were used as above. Immunohistochemical assessment: CD34 and Ki67 positive staining showed dark purple color at cell membrane and nuclear respectively, while α-SMA positive staining cardinal red in cytoplasm.

### MV number density (MVND) and microvascular pericyte number density (MPND) assessment

In counting the number of MVs, single EC as one count unit no matter if the vessel lumen had conformed or red blood cells presented inside the lumen. The remnants of vessels or the vessels with lumen diameter>8 red cells were not counted [23] [24]. Glioma CD34^+^ MVND was defined as the average number of CD34^+^ MVs per high-magnification field (x400) while defining α-SMA^+^ MVND in the same way. As the abundance and structure of MVs within glioma varied too much from location to location, the vascular hotspots were identified by screening for the areas with highest vessel density at low-power magnification. Then, a minimum of 5 non-repeat fields with high-power magnification (x400) from each section were selected to analyze vasculature. The numbers of CD34^+^ or α-SMA^+^ MVs per field were manually counted respectively, and CD34^+^ MVND and α-SMA^+^ MVND were calculated. The ratio between α-SMA^+^ MVND and CD34^+^ MVND was calculated to describe pericyte composition in MVs. Similarly, the numbers of α-SMA positive cells were also counted and expressed as MPND. All of the stained sections were evaluated separately by at least two pathologists, and their variation was less than 5% in mean values. The results were showed as Mean ±SD.

### Quantification of α-SMA positive area density in MVs

The proportion of α-SMA positive area reflected the MV pericyte area density (MPAD), a index of pericyte abundance. Following the selection of angiogenesis hotspots, 5 fields were randomly chosen to quantify the α-SMA^+^ area in MVs. Total 5 random fields from each section were captured at 1360×1024 pixel resolution under 400× magnification under Olympus BX51DP70 microscope (Olympus Microsystems, Japan). All images were captured under the same exposure settings. The uniformly magnified images were analyzed for MPAD and the integrate optical density (IOD) by Image Pro-Plus system (version 6.0, Media Cybernetics Company, Silver Spring, MD, USA) as described previously [25]-[27].

### Quantification of PDGFβ immunohistochemical staining

The PDGFβ immunohistochemical staining was first scored using a 2-tiered scoring system as semi-quantification on the staining intensity (SI) of the visualizing signal and the percentage of positive cells (PP) following the protocol previously described [28], [29] with slight modification. The SI was scored as negative (score = 0), weak positive (light yellow, score = 1), moderate positive (yellow to brown, score = 2) and strong positive (dark brown, score = 3) at the corresponding cellular location. The PP of 0, <25%, 26-50% and 51–100% were scored as 0, 1, 2 and 3, respectively. The total immunoreactive score (IRS) was calculated as follows: IRS = SI×PP, which ranged from 0 to 9, and assigned as negative (score = 0–1), positive (score = 2–4) and strong positive (score>4).Then the Image Pro-Plus was used to analyze and evaluate positive PDGFβ expression with the process as described for α-SMA expression. In each section, at least five fields (x400) were recorded and analyzed.

### Statistical analysis

The differences between different groups (tumor grades) were analyzed by using one-factor analysis of variance with Statistical Package for Social Science (SPSS for Windows, version 12.0). The correlation between the PDGFβ expression and MPND in different grade of glioma was analyzed for correlation coefficient. Two-tailed *P* values of 0.05 or less were considered to be statistically significant.

## Results

### Patients and their pathological characteristics of the microvasculature in glioma

All of the tumor tissue samples of glioma patients were collected from Institute of Pathology, Southwest Hospital in the period of 2006–2009. According the classification guidance from WHO, of the 40 tumor patients, 12 fell into grade WHO II, 12 into WHO III, and 16 into WHO IV. The clinical characteristics of patients and tumor location were shown in Table 1.

**Table 1:** The clinical characteristics of patients.

	Median Age (yrs)	Mean Age (yrs± SD)	gender		Location/involve extent		
			M	F	SA	2A	3A
**WHO II (n = 12)**	36	36.7±8.1	7	5	8	4	
**WHO III (n = 12)**	42	41.8±13.2	8	4	7	4	1
**WHO IV (n = 16)**	47	46.8±15.3	11	5	10	6	

SA, single cerebral area (lt or rt temporal/frontal/parietal lobe) involved; 2A, two cerebral areas(lt or rt temporal & frontal/parietal, frontal & parietal, parietal & occipital lobe); 3A, three cerebral areas(rt basal ganglia and temporal & frontal lobe).

**Figure 1:**
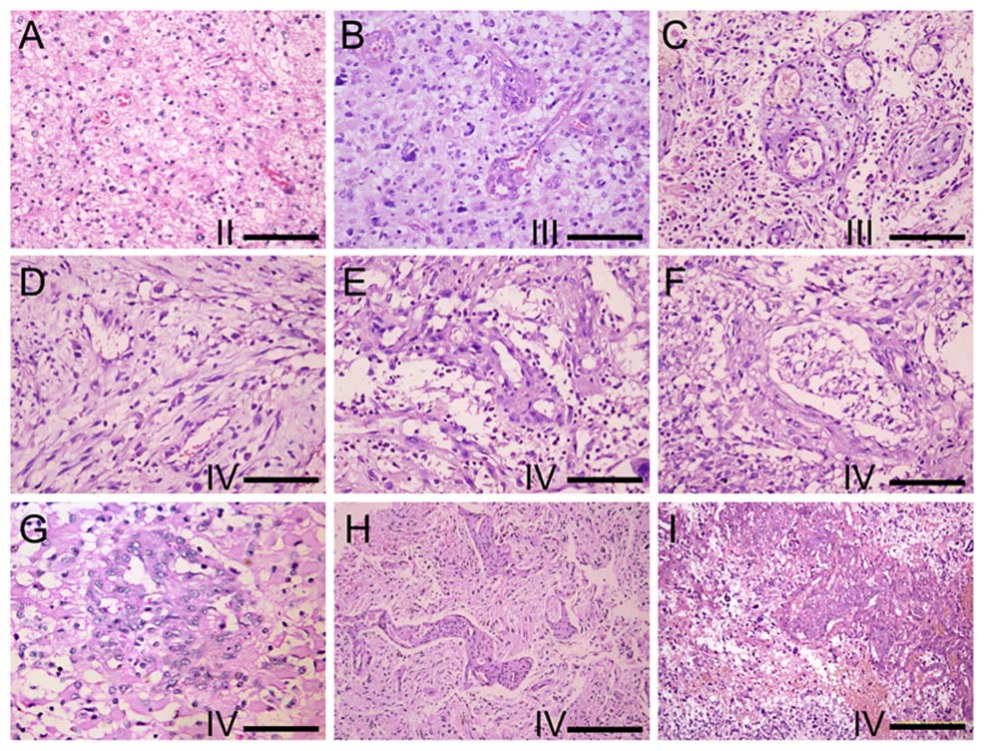
The morphology changes of glioma microvasculature along with the increase of the WHO grade. (A) thin-wall or sinusoid vessels with different sizes of lumen in grade II glioma, (B-C) irregular buds, cell cords and thick-wall vessels in some areas of grade III glioma, (D-H) strip cord, plexus, glomeruloid, ophidian microvessels in grade IV glioma and (I) more proliferation of heterotypical vessels found around necrotic and hemorrhagic areas in grade IV glioma. (HE A-G Bar = 100 um, H and I Bar = 200 um).

Generally, the MV numbers and the multiplicity and complexity of the vasculatures in tumor stroma increased with the malignant grades of glioma. Less MV number and relatively normal structure were identified in grade II glioma of WHO classification, in which often seen were the thin-wall or sinusoid vessels with different sizes of lumen (Fig. 1A). In grade III glioma, besides the thin-wall vessels as above, irregular shape vessels like buds or cell cords and thick-wall vessels appeared in some areas (Fig. 1B, C). In grade IV, the most malignant glioma, high multiplicity of microvasculature was represented by the plexus, strip cords, ophidian and glomeruloid MVs (Fig. 1D-H). In some areas, these irregular vasculatures filled up almost all of the stromal spaces. It was worthy of note that the lumen of these vessels was not enlarged and even shrunk in some vessels, and that the increase of MV area was due to the hyperplasia of vascular wall cells. This heterotypical vessel morphology was quite often in high grade glioma around necrotic and hemorrhagic areas (Fig. 1 I).

### The MV architectures in glioma of different malignant grades

**Figure 2:**
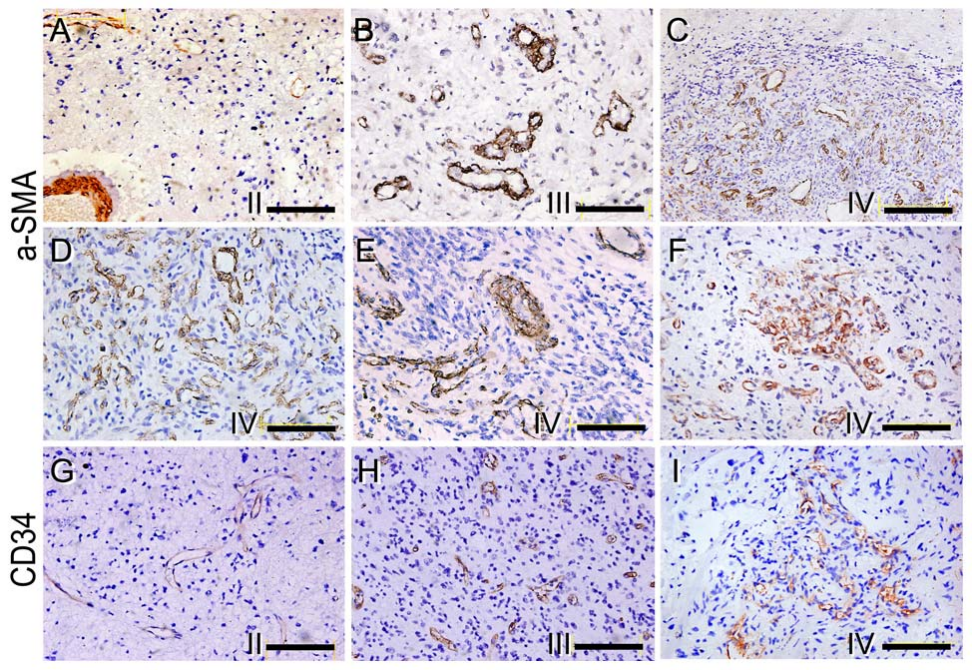
Immunohistochemical staining for α-SMA (A-F) and CD34 (G-I) of glioma. (A, G) Single layer cells in the microvessels showed either α-SMA or CD34 WHO grade II glioma. Multiple layer of vessels cells were α-SMA positive while only the lining cells of the vessels were CD34 positive in grade III (B, H) and grade IV (C-F, I) glioma (Bar = 200 um in C, Bar = 100 um in the rest).

Immunohistochemical staining positioned pericyte marker α-SMA to cell cytoplasm, and the positive cells located outside the vessels and around ECs (Fig. 2A-F). The EC marker CD34 was mainly expressed at cell membrane, and the positive cells resided at the lining layer of MVs (Fig. 2G-I). This was coincidence with the known location of the ECs and the pericytes. It was revealed that the amount of α-SMA positive cells, the pericytes, was even higher than that of CD34-positive ECs in high grade glioma. The main cell composition of the thin-wall MVs in WHO grade II was CD34 positive ECs with a few α-SMA positive cells around (Fig. 2A, G). The same cell constitution was also found in the thin-wall MVs in high grade glioma. But, in the wall of thick-wall MVs, multilayer of α-SMA positive cells was identified (Fig. 2B). Remarkably, no matter the multiformity of MVs in WHO grade IV, only the monolayer lining cells exhibited positive staining of endothelial cell marker. Meanwhile, most of the cell components in MV areas showed α-SMA positive (Fig. 2I,C-F).

**Figure 3:**
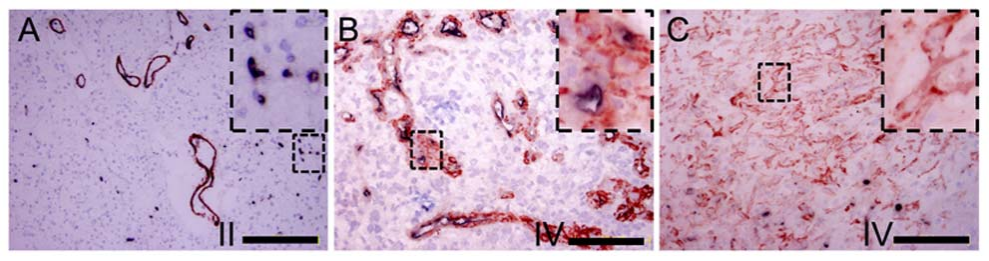
Immunohistochemical double staining against CD34 and α-SMA. (A) The inner layer of thin microvessels showed CD34 dark purple staining while the closely connected outer layer was in α-SMA red staining in WHO grade II glioma (Bar = 200 um). (B) Multiple layer of microvessels cells were α-SMA positive while only the lining cells of the vessels were CD34 positive. (C) In some areas of WHO grade IV glioma, focal, tubular or cordal cell clusters were found to be α-SMA positive only.(Bar = 100 um).

Double staining using CD34 and α-SMA antibodies confirmed the expression of these two molecules in closely arranged ECs and pericytes of gliomal MVs. The CD34 positive ECs in dark purple confined to the vessel lining while the red pericytes to the outside layer around (Fig. 3). Majority of the thin-wall vascular cells showed dark purple in WHO grade II and higher grades (Fig. 3A). With the increase of the thickness of vessel wall, more red stained cells appeared enclosing the lining cells in WHO grade II and IV, especially at the thick-wall, plexus and glomeruloid MVs (Fig. 3B). Furthermore, in some areas, focal, tubular or cordal cell clusters were found to be α-SMA positive only, indicating the possible guiding role of pericytes in the angiogenic process in the high grade glioma (Fig. 3C).

**Figure 4:**
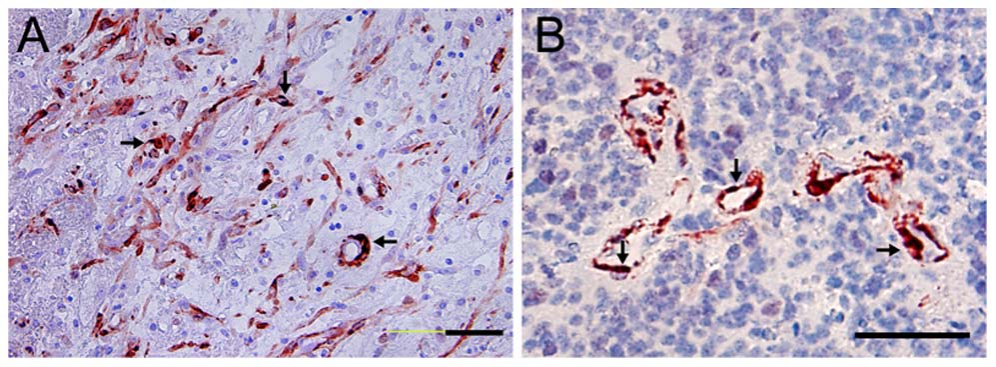
Immunohistochemical double staining Ki67 and α-SMA. The microvessel cells showed α-SMA positive (red in plasma) while some of the cells were also Ki67 positive (dark purple in nuclear as indicated by arrows) in WHO grade IV glioma (Bar = 100 um).

Double staining with Ki67 and α-SMA antibodies showed most nuclear ki67-positive vessel cells coexpressed α-SMA in high grade glioma, particularly at the thick-wall MVs (Fig. 4A,B) and the pericytes around the zone of necrotic or hemorrhagic areas in WHO grade IV glioma(Fig. 4A). The results provided evidence of pericyte proliferation in high grade glioma and the source of hyperplastic pericytes, e.g., the proliferation of local pericytes contributed to the hyperplastic phenotype.

### Quantitative analysis of MV constitution in glioma

The numbers of MVs indicated by EC marker CD34 or pericyte marker α-SMA, and the number of pericytes by α-SMA staining were counted respectively for the calculation of the microvessel number density and the MV pericyte number density, i.e. MVND and MPND. Both of the parameters showed a trend of augmentation as the WHO grade increased. The CD34^+^-MVND was 14.90±4.67, 25.83±12.73 and 29.41±18.06 in grade II, III and IV, respectively, and the differences between groups were statistically significant (*P*<0.01) (Table 2) except between grade III and IV. The α-SMA^+^-MVND was 5.30±3.07, 12.89±6.01 and 22.45±14.72 in grade II, III and IV, respectively and there were significant differences between groups (*P*<0.01). That α-SMA^+^MVND was lower than CD34^+^ MVND indicated part of the MVs was consisted of ECs only. The α-SMA^+^MVND/CD34^+^ MVND ratio increased from 35.57% in grade II to 76.36% in grade IV (Table 2), suggesting the preferential proliferation of pericytes and more pericyte-contained MVs in high grade glioma.

**Table 2:** MVND and MPND analysis in glioma.

	WHO II (n = 12)	WHO III (n = 12)	WHO IV (n = 16)
**CD34^+^-MVND**	14.90±4.67	25.83±12.73^a^	29.41±18.06^a^
**α-SMA^+^-MVND**	5.30±3.07	12.89±6.01^a^	22.45±14.72^ab^
**α-SMA^+^/CD34^+^ MVND (%)**	35.57	49.90	76.36
**MPND**	8.65±4.45	37.08±28.79^a^	81.61±69.85^ab^

a, *P*<0.01 vs grade II; b, *P*<0.01 vs grade III.

MVND, microvessel number/400 x field; MPND, α-SMA^+^ cell number in microvessel/400 x field.

The MV pericyte number density (MPND) also showed rising tendency with the increase of glioma grade, and significant differences between each of the groups were proved (*P*<0.01) (Table 2). A huge increase of MPND in grade IV was identified, which was more than two-fold of that in grade III.

**Figure 5:**
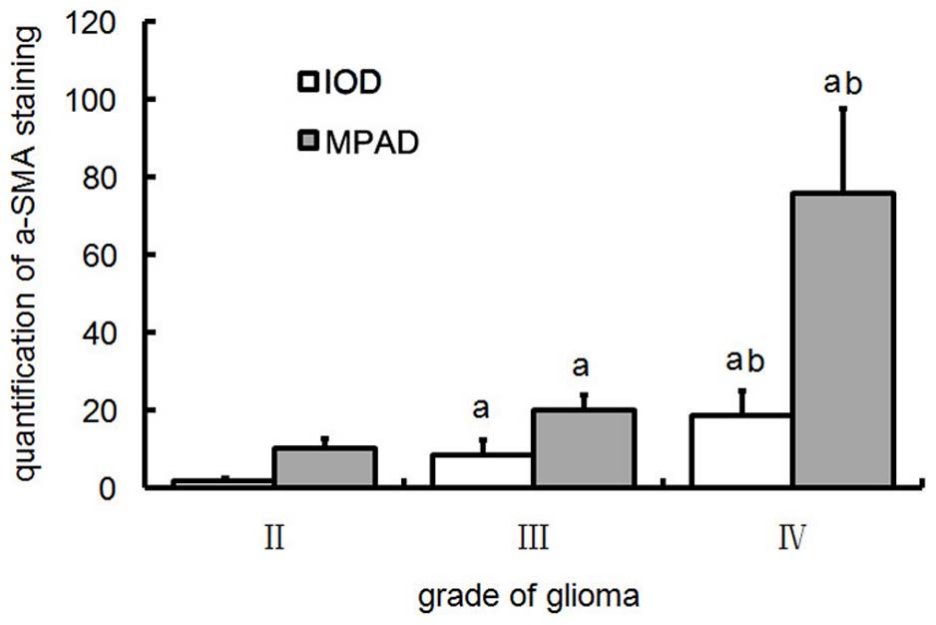
Quantitative analysis of α-SMA positive microvessels in glioma. a, P<0.05 vs grade II; b, P<0.01 vs grade III.

The images of α-SMA staining were also analyzed by image processing software in term of MV pericyte area density (MPAD) and integrate optical density (IOD). With the rising of WHO grade of glioma, both of the MPAD and IOD ascended, indicating the increase of pericyte-occupied area and the staining intensity. There were obvious differences between different grade groups (Fig. 5). All of the above results demonstrated that the preferential proliferation of pericytes with the increase of glioma grades was a pathological hallmark.

### The expression of PDGFβ and its relationship with MPND and grade of glioma

**Figure 6:**
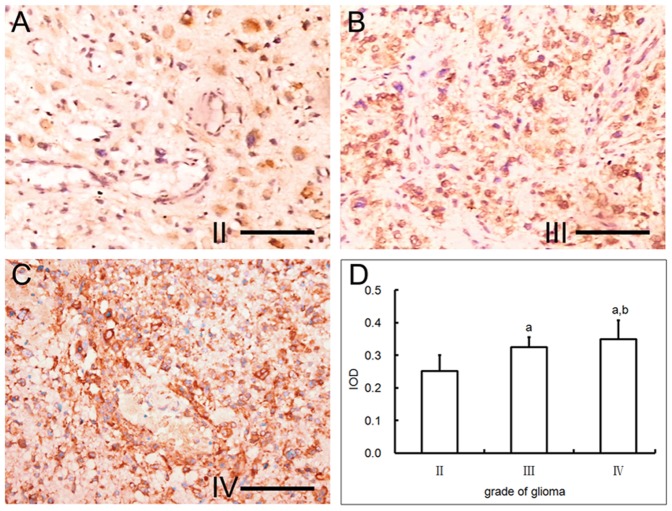
PDGFβ expression and quantification in glioma. Immunohistochemical staining against PDGFβ in WHO grade II (A), III (B) and IV (C) glioma(Bar = 100 um). (D) Quantitative analysis of PDGFβ staining results in different grade glioma. a, P<0.01 vs grade II; b, P<0.05 vs grade III.

The positive immunostaining for PDGFβ, which are usually produced by tumor cells, showed yellow to brown color in cytoplasm. The extent and cell number of PDGFβ expression were augmented with the increase of glioma grades (Table 3). Commonly, the expression mode exhibited as that local or focal positive staining with weak to moderate intensity in grade II glioma (Fig. 6A), and more extensive and stronger in higher grades (Fig. 6B and 6C). Quantification of PDGFβ immunostaining results showed that IOD was significantly different between each groups (*P*<0.05) (Fig. 6D). It should be mentioned that robust expression of PDGFβ was identified at the peripheral areas around necrotic, hemorrhagic and edematous foci. The expression of PDGFβ was correlated with the MPND of glioma, and the coefficient correlation was 0.914. It was interesting that the expression of PDGFR-β was also found in pericytes in addition to the known tumor cells.

**Table 3:** PDGFβ expression in glioma.

	negative		Positive	Strong positive	Positive rate (%)
**WHO II (n = 12)**		4	8	0	66.7
**WHO III (n = 12)**		2	7	3	83.3
**WHO IV (n = 16)**		2	8	6	87.5

## Discussion

Tumor angiogenesis is a very important step in tumor growth and progression. The nascent MVs constitute the majority of tumor stroma. It is believed that tumor blood vessels possess the characteristics of disordered architecture and abnormal function [10], [30]. For example, they are more dilated and tortuous, with excessive branching, lack of the normal artery-capillary-vein hierarchy, and increased cellular fenestrations as well as widened intercellular junctions or gaps. These abnormal structures bring about high permeability and hyperosmosis in tumor stroma, and further may lead to hypoxia, acidity, insufficient nutrient supply, and resistance to antitumor drug delivery and even chemoradiotherapy [9], [31]. For a long time, research attention has not been paid to tumor vasculature because the pathological changes of tumor vessels are believed to be lack of specificity. Actually, specific features of vessels in tumor may exist among different tumors [7], [10]. Firstly, vasculature may be formed by different processes or sources like angiogenesis and vasculogenesis; secondly, different tissues or organs have their special vessel features that are the tissue-specific manner; Thirdly, at different stages, the newly formed vessels have their own different features. Nagy et al described that mother vessels and glomeruloid microvascular proliferations are common at early stage, while at late stage, the formation of capillaries and vascular malformations by angiogenesis, and feeder arteries and draining veins by arterio-venogenesis [32]. All of the above factors contribute to the vascular heterogeneity in and among tumors. It is suggested that tumor microvasculature heterogeneity does exist, and may be one of the main factors influencing tumor growth, therapeutic efficacy and prognosis, highlighting a novel research field in oncology. In recent years, the concept of tumor microvascular architecture phenotype (T-MAP) has been proposed and tested in glioma as well as in other types of tumors [6], [11]. T-MAP describes the characteristics of nascent microvasculature in the aspects of shape, architecture and three-dimensional distribution in tumor tissue. T-MAP may exhibit multiple diversity and variability along with the heterogeneity of tumor cells.

The classical characteristics of microvascular pathology in tumor stroma are the hyperplasia of ECs accompanied by hemorrhage, necrosis and edema, which is one of the important diagnostic criteria for malignant glioma, particularly the Glioblastoma multiforme (GBM). It is commonly accepted that the MVs in tumor stroma and its derivative MVND increased consistently with the malignant grade of glioma [20], [33] In previous study, we found that the hyperplastic vascular cells did not necessarily express EC markers. There was no hyperplasia of the lining ECs in thick-wall, plexus and glomeruloid microvascular proliferation. Instead, the number of cell layer in vessel wall was increased with disordered cell alignment. In present experiment, using multiple markers to probe ECs and pericytes, we found that most of the microvascular cells displayed pericyte marker α-SMA/PDGFRβ while the EC marker only confined to the monolayer lining cells, suggesting that pericytes not ECs are the main cells constitute the multiplicity of MVs. Similar phenomenon was also found in dedifferentiated chondrosarcoma, higher pathologic grades of clear cell renal cell carcinoma (CCRCC) and other malignant tumors [8], [34]. In 2 cohorts CCRCC researches from Asian and US, Cao et al even found that higher amount of pericytes was correlated with more aggressive clinicopathological characteristics, such as more advanced tumor classification, higher pathological grades and tumor necrosis, suggesting pericyte was an independent unfavorable prognostic factor. As one of the key components of vasculature, pericytes may play an important role in determining the different pattern of T-MAP in glioma.

Normally pericytes are flat cells with prominent cell protrusions and surround ECs in capillaries, those two types of cells are usually connected by basement membrane in between as well as cell-cell junctions, which may control their interaction. It is believed that the ECs are the leading cells in angiogenesis, while pericytes only take part in the blood vessel modeling and maintaining vessel stability. The pericyte labeling index is used to be a parameter to indicate the degree of MV maturity. Although pericytes have been found for more than 100 years, research on pericytes was significantly lagged behind that of the ECs, especially on their roles in angiogenesis. The main obstacles are the heterogeneicity in gene expression and distribution, and the lack of special marker. Recently, with the identification of some high specific pericyte markers, such as α-SMA, the regulator of G-protein signaling-5 (RGS5), nerve/glial antigen 2 (NG2) and PDGFR-β, more progress has been achieved in understanding the physiological and pathophysiological roles of pericytes in vascular development and maintenance. Our research confirmed that α-SMA is a good marker of pericytes to show the pericyte composition of MVs in glioma, which is consistent with Cao's result using α-SMA to allocate pericytes in the MVs of CCRCC [34]. Some researchers suggested that pericytes play a guiding role at the early time of angiogenesis, for example, pericytes may perceive the angiogenic stimuli and guide the out-growth of vessel buds as the pioneer of angiogenesis [16], [35]. Recent research found that glioblastoma stem cells (GSCs), which have the pivotal potential to drive tumorigenesis, metastatic growth, recurrences, and even treatment resistance, not only could transdifferentiate into ECs but also into pericytes (G-pericyte), and the latter took larger proportion. Targeting of G-pericytes may block tumor progression and effectively improve anti-glioblastoma therapy [18], [19]. This finding not only revolutionarily turns our belief on pericytes from the subsidiary role to the leading role in angiogenesis, but also proposes a new strategy to target pericytes for interfering angiogenic process [17]–[19]. Our observation confirmed for the first time that pericyte-only structures in malignant glioma can be found at the new vascular buds, cell cords, tubules, and hyperplastic pericytes in the wall of heterotype vessels that commonly were immature MVs. The existence of pericyte-only structures suggests that pericytes may, as the leading cells, initiate angiogenesis and perform vessel function like ECs, and this may also be applied to normal blood vessel development as a complementary mechanism if not solely.

In present study, several methods were applied to quantitatively and qualitatively measure the amount of pericytes in MVs of glioma. The conclusion of hyperplasia of pericytes in glioma was drawn based on follows: with the advance of malignant grades of glioma, the structure of MVs displays abnormal pattern, such as plexus, cordal, thick-wall, and glomeruloid MVs. CD34 positive cells presented only in the lining layer while α-SMA in the single to multiple outside layers of cells. The α-SMA^+^ MVND, MPND, and MPAD consistently showed the increase of pericytes in number and occupied area. Ki67 staining prompted the proliferation of local pericytes may be the source of cell hyperplasia.

The course of angiogenesis was controlled by multi-angiogenic factors like VEGF, PDGF, TGF-β and bFGF. VEGF is the main factor that controls the migration, proliferation and survival of ECs. However, PDGFβ mainly impacts on pericytes and vascular smooth muscle cells [15], [36]. The high expression of PDGFβ in glioma was related with the progression and angiogenesis of the tumor [37]. In our study, with the increase of WHO grades, the expression of PDGFβ was upregulated, and well correlated with the proliferation of pericytes in glioma, especially in grade IV. In the hemorrhagic and necrotic areas of tumors, the local PDGFβ level was notably high, which in turn may induce the differentiation of pericyte progenitors, the migration and proliferation of pericytes to potentiate angiogenesis. These remarkable responses of pericytes to PDGFβ could result in the hyperplasia of pericytes and the multiplicity of MV formation in glioma. It also suggests the increase of PDGFβ may be one of the main causes for the formation of heterogeneous MV structure [38].

It should be mentioned that the CD34^+^-MVND was not correspondingly increased in WHO grade IV glioma when compared with that in grade III, while the α-SMA^+^ MV area was obviously enhanced in this research. It can be explained by the preferential hyperplasia of pericytes other than ECs in the thick-wall vessels, which took more space instead of increasing vessel number as well as the MVND. In this circumstance, we suggest replacing MVND with MV area, MPND or MPAD as an indicator for vessel hyperplasia.

## Conclusions

Present study demonstrated that the hyperplastic pericytes are one of the important constituents of heterogeneous MVs in higher pathological grade glioma especially in GBM, and directly affect tumor microvascular architectures. The existence of pericyte-only structures, like vessel buddings and vessel-like structures suggest that pericytes may play an important role in angiogenesis as guiding cells. In glioma of high grade as well as the one with hemorrhage and necrosis, the high expression of PDGFβ could be a main cause for the hyperplastic response of pericytes. In considering that pericytes participate the construction and influence the architecture of MVs in glioma, pericytes could be a new target for the development of pro-angiogenetic and anti-angiogenetic therapies.
